# Integrating bioinformatics and machine learning to identify biomarkers of branched chain amino acid related genes in osteoarthritis

**DOI:** 10.1186/s12891-025-08779-6

**Published:** 2025-05-26

**Authors:** Xiao-Zhi ZhaYang, Yan-Xiong Chen, Wen-Da Hua, Zheng-Lin Bai, Yun-Peng Jin, Xing-Wen Zhao, Quan-Fu Liu, Zeng-Dong Meng

**Affiliations:** 1 Faculty of Medical Science, Kunming University of Science and Technology, Kunming, Yunnan China; 2 Department of Orthopedic Surgery, The First People’s Hospital of Yunnan Province, Affiliated Hospital of Kunming University of Science and Technology, Kunming, Yunnan China; 3 Yunnan Key Laboratory of Digital Orthopedics, Kunming, Yunnan China; 4 Department of Orthopedic Surgery in The First People’s Hospital of Yunnan Province, Affiliated Hospital of Kunming University of Science and Technology, No.157, Jinbi Road, Xishan District, Kunming, Yunnan 650032 China

**Keywords:** Osteoarthritis, Branched-chain amino acids, Machine learning, Bioinformatic analysis, Drug prediction

## Abstract

**Background:**

Branched-chain amino acids (BCAA) metabolism is significantly associated with osteoarthritis (OA), but the specific mechanism of BCAA related genes (BCAA-RGs) in OA is still unclear. Therefore, this research intended to identify potential biomarkers and mechanisms of action of BCAA-RGs in OA tissues.

**Methods:**

Differential genes were obtained from the Gene Expression Omnibus (GEO) database and intersections were taken with BCAA-RGs to identify candidate genes. The underlying mechanisms were revealed using Gene Ontology (GO) and Kyoto Encyclopedia of Genes and Genomes (KEGG). Subsequently, by combining three machine learning algorithms to identify genes with highly correlated OA features. In addition, created diagnostic maps and subject Receiver operating characteristic curves (ROCs) to assess the ability of the signature genes to diagnose OA and to predict their possible roles in molecular regulatory network axes and molecular signaling pathways.

**Results:**

Eight candidate genes were acquired by intersecting 4,178 DEGs and 14 BCAA-RGs. Subsequently, five candidate biomarkers were obtained, namely SLC3A2, SLC7A5, SLC43A2, SLC43A1, and SLC7A7. Importantly, SLC3A2 and SLC7A5 were validated by validation set and qRT-PCR. Furthermore, the nomogram constructed by SLC3A2 and SLC7A5 exhibited excellent accuracy in predicting the incidence of OA. The enrichment results demonstrated that SLC3A2 and SLC7A5 were significantly enriched in ribosome, insulin signaling pathway, olfactory transduction, etc. Meanwhile, we also found XIST regulated SLC7A5 through hsa-miR-30e-5p, and regulated SLC3A2 through hsa-miR-7-5p.OIP5-AS1 regulated SLC7A5 and SLC3A2 through hsa-miR-7-5p. By the way, 150 drugs were identified, including Acetaminophen and Acrylamide, which exhibited simultaneous targeting of these two biomarkers.

**Conclusion:**

Based on bioinformatics, SLC3A2 and SLC7A5 were identified as biomarkers related to BCAA in OA, which may provide a new reference for the treatment and diagnosis of OA patients.

**Supplementary Information:**

The online version contains supplementary material available at 10.1186/s12891-025-08779-6.

## Introduction

Osteoarthritis (OA) is a common chronic joint disease that involves multiple tissues, including articular cartilage, synovium, and subchondral bone [1, 2]. It is estimated that over 500 million people worldwide suffer from OA, with a higher prevalence in the elderly population [3]. The etiology of OA is remains incompletely understood, but it is thought to involve multifactorial processes. Possible pathogenic mechanisms include degenerative changes in joint cartilage, arthritic inflammation, and the cumulative impact of biomechanical factors [4–6]. These factors lead to structural damage and functional impairment of the joints, triggering pathological processes. Joint pain and functional impairments not only affect daily activities but may also lead to loss of productivity, increased medical expenses, and rehabilitation costs [7, 8]. Despite the availability of some diagnostic and treatment methods for OA, such as clinical symptom assessment, imaging studies, and medication, there are still challenges and issues. These include difficulties in early diagnosis, inconsistent treatment outcomes, and the lack of individualized treatment plans [9, 10].Therefore, there is an urgent need to find more scientific and effective approaches for early diagnose and treatment of OA.

BCAAs, comprising leucine, isoleucine, and valine, are essential amino acids with distinctive branched molecular structure [11, 12]. BCAAs have important physiological functions in the human body, including involvement in protein synthesis, providing energy, and regulating insulin secretion, among others [13, 14]. BCAAs have been shown to modulate key metabolic and signaling pathways, influencing overall metabolic homeostasis and cellular function. Emerging evidence highlights a potential link between BCAA levels and the development of OA and its related diseases. Some clinical and epidemiological studies suggest that BCAA levels are associated with the occurrence of osteoarthritis, pain severity, and disease progression [15]. Additionally, elevated BCAA levels are also associated with the onset and progression of diseases such as obesity, metabolic syndrome, and diabetes [16, 17]. BCAA is associated with the upregulation of key pro-inflammatory cytokines implicated in OA pathophysiology, which primarily leads to the degradation of articular cartilage matrix [18]. However, despite the discovered association between BCAAs and osteoarthritis and other diseases, the specific mechanism of action of BCAAs in OA remains unclear. Recent studies have demonstrated significant potential of machine learning in OA prediction, particularly for diagnosing and predicting the progression of knee osteoarthritis [19]. Machine learning algorithms have been successfully applied to analyze clinical data, such as peripheral blood DNA methylation levels, as well as imaging modalities like X-rays and ultrasound [20]. Incorporating machine learning into OA research thus provides a powerful tool for enhancing patient outcomes and advancing our understanding of OA pathogenesis.

Based on the important role of BCAAs in the development of OA, we will further understand the role of BCAAs-related genes in the regulation of amino acid function and metabolic progression, so as to provide some reference value for the pathogenesis and diagnosis of OA. Therefore, we used osteoarthritis-related transcriptome data in the GEO database by bioinformatics methods to identify biomarkers associated with BCAAs, and established a diagnostic model based on branched-chain amino acid-related pathways using machine learning methods in order to more accurately diagnose and predict the pathogenesis of OA. In addition, we further investigated the potential biological functions and molecular mechanisms associated with these signature genes using Gene Ontology (GO)/Kyoto Encyclopedia of Genomes (KEGG) enrichment analysis and Genome Enrichment Analysis (GSEA) and performed drug prediction analysis to identify the most relevant potential drugs. Our findings may provide new insights into the pathogenesis of OA and contribute to the discovery of new biomarkers for improved diagnosis and therapeutic outcomes.

## Materials and methods

### Data extraction

**Fig. 1:**
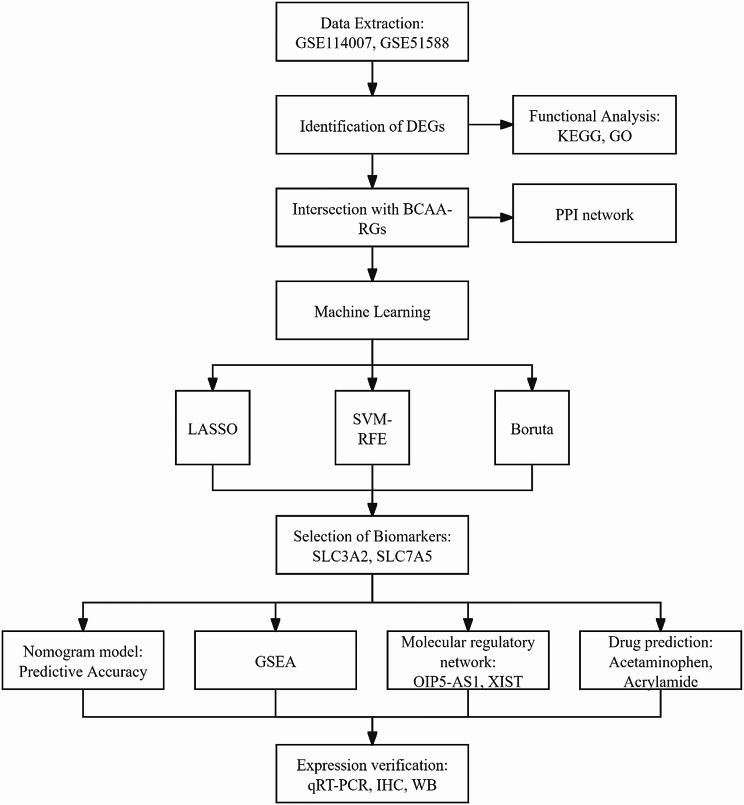
The flowchart of this study

Osteoarthritis (OA) related datasets (GSE114007 and GSE51588) were provided by the Gene Expression Omnibus (GEO) (http://www.ncbi.nlm.nih.gov/geo/) database. The GSE114007 dataset was used as a training set with 38 samples, including 18 normal cartilage tissue samples and 20 OA cartilage tissue samples. The sequencing platform for this dataset was Illumina HiSeq 2000 (Homo sapiens).The annotation files for this dataset were GPL11154 and GPL1857. In this dataset, FastQC (v0.10.1) was used to perform quality control of the raw data on the fastq files. The raw data were aligned with the human genome (hg19) using the STAR aligner v(2.5.3a), and the aligned reads were quantified using HTSeq-count with the UCSC RefSeq hg19 annotation (Release 57). The validation set, GSE51588, comprised 50 samples (10 normal and 40 OA cartilage tissue samples). For this dataset, the microarray platform used was Agilent-026652 Whole Human Genome Microarray 4 × 44 K v2 (Probe Name version). The annotation file for this dataset was GPL13497. In this dataset, the array signal intensities were further analyzed by the Agilent GeneSpring GX software (Version 11.5) (Agilent Technologies, Santa Clara, CA, USA). The gene expression values of all datasets were normalized using the quantile normalization method; the filter was set to above 32 to exclude probes with low signal intensities. In addition, totally 14 Branched-chain amino acids related genes (BCAA-RGs) were gained from the Molecular Signatures Database (MSigDB) (https://www.gsea-msigdb.org/) by “searching gobp branched chain amino acid transport”. The entire workflow of this study was shown in Fig. 1.

### Identification and functional enrichment analysis of differentially expressed genes (DEGs)

The sample distribution of the GSE114007 dataset was analyzed by principal component analysis (PCA) to determine whether there were outliers that needed to be eliminated, thus ensuring the reliability of the data analysis. Subsequently, based on the screening conditions of|log2FoldChange(FC)| > 0.5 and P adj < 0.05, the DESeq2 package (v 1.42.0) [21]was used to compare the differences of gene expression between OA and control groups to obtain DEGs. The expression of DEGs was demonstrated by plotting volcano and heat maps with the ggplot2 package (v 3.3.6) [22]and the “pheatmap” package (v 1.0.12) [23], respectively. In particular, the heat map displayed the|log2FC| top 40 DEGs. In addition, Gene Ontology (GO) function and Kyoto Encyclopedia of Genes and Genomes (KEGG) function and pathway enrichment analyses were conducted. The GO enrichment analysis included the analysis of molecular function (MF), cellular component (CC), and biological process (BP). The purpose was to find the common functions and enrichment pathways (P adj < 0.05) of the differentially expressed genes (DEGs) through the clusterProfiler software package (v 4.4.4) [24] We further conducted enrichment analyses on the upregulated DEGs and downregulated DEGs respectively in order to obtain more meaningful biological interpretations.

### Screening of candidate biomarkers

Candidate genes associated with both OA and BCAA-RGs were obtained by taking intersections of DEGs with BCAA-RGs. Subsequently, candidate genes were analyzed for the construction of protein-protein interaction (PPI) network based on the STRING database (https://string-db.org/) (degree of reciprocity threshold = 0.4), thus exploring the existence of reciprocal relationships between these genes.

### Machine learning

In GSE114007 dataset, the expression of candidate genes was analyzed by least absolute shrinkage and selection operator (LASSO) using glmnet package (v 4.1-4) [25] to get the feature genes of this algorithm, among them, the parameters were set as family = “binomial” and nfolds = 10. The support vector machine recursive feature elimination (SVM-RFE) algorithm was also performed using the e1071 package (v 1.7–14) [26]thus obtaining the importance and importance ranking of each candidate gene, as well as obtaining the error rate and accuracy of each iteration of the combination, selecting the lowest point of the error rate as the best combination, and taking out the corresponding genes as the feature genes. The parameter “kernel” was set as “linear” during this process. In addition, Boruta algorithm was implemented for screening candidate genes through Boruta package (v 8.8.0) [27], among them, the criteria were set as pValue = 0.05 and maxRuns = 100. Furthermore, candidate biomarkers were obtained by overlapping the feature genes obtained from the three machine learning algorithms.

### Candidate gene expression and diagnostic capability assessment

In the GSE114007 and GSE51588 datasets, the expression of candidate genes was evaluated between OA and control groups, while receiver operating characteristic (ROC) curves were plotted via pROC package (v 1.18.0) [28] to assess the diagnostic ability of the candidate biomarkers. Candidate biomarkers with consistent expression trends in the two datasets, significant differences between OA and control groups, and area under the curve (AUC) ≥ 0.7 were defined as biomarkers.

### Construction and evaluation of nomogram

In order to more intuitively understand the relationship between biomarkers and OA, a nomogram was constructed based on multivariate logistic regression through rms package (v 6.5.0) [29]. The corresponding scores were obtained by detecting the expression of each biomarker, and then the incidence of OA was judged according to the total score. To further explore the relationships among the two key genes, clinical characteristics (such as age and gender), and osteoarthritis (OA), another nomogram was constructed in this study based on multiple logistic regression. The corresponding scores were obtained by detecting the expression levels of each key gene. Meanwhile, factors such as age and gender in the clinical information were combined to calculate the corresponding scores, and the incidence rate of OA was evaluated according to the total scores.

Moreover, the calibration curve and ROC curve were adopted to verify the accuracy of the nomogram prediction.

### Gene set enrichment analysis (GSEA)

With the aim of further exploring some relevant signaling pathways and potential biological mechanisms present in the biomarkers, GSEA was performed on the biomarkers using the clusterProfiler package and the org. Hs.eg. db package (v 3.15.0) [30]. Specifically, GSEA was implemented by ranking all genes based on the correlation coefficients between the biomarkers and the expression of all genes in the GSE114007 dataset. The thresholds were set to|normalized enrichment score (NES)| > 1, *Nominal P* < 0.05, and *q* < 0.25.

### Molecular regulatory network construction

The Starbase database (https://ngdc.cncb.ac.cn/databasecommons/database/id/169) was utilized to predict biomarker-associated microRNAs (miRNAs) and long noncoding RNAs (lncRNAs). The miRNAs were selected based on their prediction in both the miRanda (http://mirtoolsgallery.tech/mirtoolsgallery/node/1055) and PITA.

(http://genie.weizmann.ac.il/pubs/mir07/mir07_data.html) databases. Subsequently, clipExpNum ≥ 20 was employed as the criterion to predict miRNA-associated lncRNAs. A lncRNA-miRNA-mRNA network was constructed based on the predicted miRNAs, lncRNAs, and mRNAs.

### Drug prediction and gene-disease association analysis

The drugs targeting each biomarker were obtained through the Comparative Toxicogenomics Database (CTD) (http://ctdbase.org/). Subsequently, the predicted drugs were taken to intersection to identify key drugs that targeted the biomarkers simultaneously. Key drugs and biomarkers were selected, and the drug-gene network was constructed by Cytoscape software (v 3.9.1) [31]. On the other hand, diseases associated with biomarkers were predicted by DOSE package (v 3.82.0) [32], and a gene-disease network was constructed.

### Quantitative real-time polymerase chain reaction (qRT-PCR)

The cartilage samples obtained from patients undergoing total knee arthroplasty due to end-stage osteoarthritis were classified as OA group, and cartilage samples collected from patients without osteoarthritis who underwent distal femoral tumor resection and prosthetic replacement of the knee due to femoral tumor were classified as control group. Each group consisted of 10 samples. Total tissue RNA was extracted using TRIzol reagent (Thermo Fisher Scientific, USA) according to the manufacturer’s instructions. The purity and concentration of total RNA were detected. Subsequently, the RNA was reverse-transcribed into cDNA using Prime-Script ™ first strand cDNA synthesis kit (Thermo Fisher Scientific, USA). The synthesis was completed using PrimeScript™RT Master Mix (Takara Bio, USA). The amplification reaction was then established in a real-time PCR system using TB Green^®^Premix Ex Taq (Takara Bio, USA). GAPDH was used as an internal reference, and the 2^−ΔΔCT^ method was employed for relative quantitative analysis. The corresponding qRT-PCR primer sequences are shown in (Supplementary Table S1).

### Immunohistochemistry (IHC)

Immunohistochemical staining was performed to detect the expression of SLC3A2 and SLC7A5 in cartilage samples. Tissues were decalcified in EDTA solution at 25–30 °C in a constant-temperature shaker with solution changes every 2–3 d. After complete decalcification, samples were fixed in 4% paraformaldehyde (CNAB035-Q, Guangfu) for 24–48 h, dehydrated in graded ethanol (GB/T678-2002, Chengdu Kelon Chemicals), cleared with xylene (33535, Xilong Chemical Co.), embedded in paraffin, and sectioned into 3 μm-thick slices using a microtome (LEICA RM2135, Shuwei Instruments). Sections were deparaffinized in xylene, rehydrated through graded ethanol, and subjected to antigen retrieval in citrate buffer (G0001-1 L, Saveen) using a pressure cooker. Endogenous peroxidase activity was blocked with 3% H_2_O_2_, and non-specific binding was blocked using 5% bovine serum albumin. Sections were incubated overnight at 4 °C with primary antibodies against SLC3A2 (bs-6659R, Bioss, 1:50) and SLC7A5 (GB114871, Servicebio, 1:100), followed by a 30-min incubation at 37 °C. After PBS washes, sections were treated with a reaction enhancer (PV-9000, ZSGB-BIO) and HRP-conjugated secondary antibody (PV-9000, ZSGB-BIO), both incubated at 37 °C for 20 min. DAB substrate (ZLI-9019, ZSGB-BIO) was applied for color development, and nuclei were counterstained with hematoxylin (G1004-100ML, Saveen), differentiated with ethanol-hydrochloric acid, and blued in running tap water. Finally, sections were dehydrated, cleared, and mounted with neutral resin (WG10004160, Saveen). Staining was visualized using a slide scanner (SQS-12P, Shenzhen Qiangsheng Technology Co., Ltd.), and the expression levels of SLC3A2 and SLC7A5 were evaluated.

### Western blot (WB)

Western blot was performed to evaluate the protein expression levels of SLC3A2 and SLC7A5 in cartilage samples. Proteins were extracted using RIPA lysis buffer (G2002-30ML, Servicebio) supplemented with protease inhibitors (PR20032, Proteintech). Protein concentrations were determined through BCA protein assay kit (P0009, Beyotime). Samples were denatured by mixing with 5× protein loading buffer (G2013-100ML, Servicebio) and heating at 95 °C for 10 min. Protein separation was carried out using SDS-PAGE with a 10% gel for the resolving gel and a 5% for the stacking gel. Electrophoresis was performed at 80 V for 30 min, followed by 120 V for approximately 90 min, until the bromophenol blue dye reached the bottom of the gel. Proteins were then transferred onto PVDF membranes (0.45 μm, K2MA8350E, Millipore) using a semi-dry transfer system (Trans-Blot Turbo System, Bio-Rad) at 300 mA for 1 h at 4 °C. The membranes were blocked with 5% BSA (CR2302110, Solarbio) in TBST for 30 min at 37 °C and incubated overnight at 4 °C with primary antibodies, including anti-SLC3A2 (bs-6659R, Bioss, 1:1000), anti-SLC7A5 (GB114871, Servicebio, 1:1500), and anti-β-actin (66009-1-Ig, Proteintech, 1:25000). After washing with TBST, membranes were incubated for 60 min at room temperature with HRP-conjugated goat anti-rabbit IgG (GB23303, Servicebio, 1:3000) or goat anti-mouse IgG (GB23301, Servicebio, 1:5000) diluted in 5% skimmed milk (EZ7890B383, BIO FROXX). Membranes were washed with TBST and developed using ECL substrate (KF8001, Affinity). Signals were captured using a chemiluminescence imaging system (XLSY153, Qinxiang). The intensity of protein bands was quantified using ImageJ software (version8.0, NIH, USA) for grayscale analysis.

### Statistical analysis

R software (version 4.2.1) was used to process and analyze the data. The correlation analysis between the two groups was implemented via Spearman analysis. Differences between the two groups were compared via Wilcoxon test. The p value less than 0.05 was considered statistically significant.

## Results

### Totally 4,178 DEGs were related to multiple signaling pathways

**Fig. 2:**
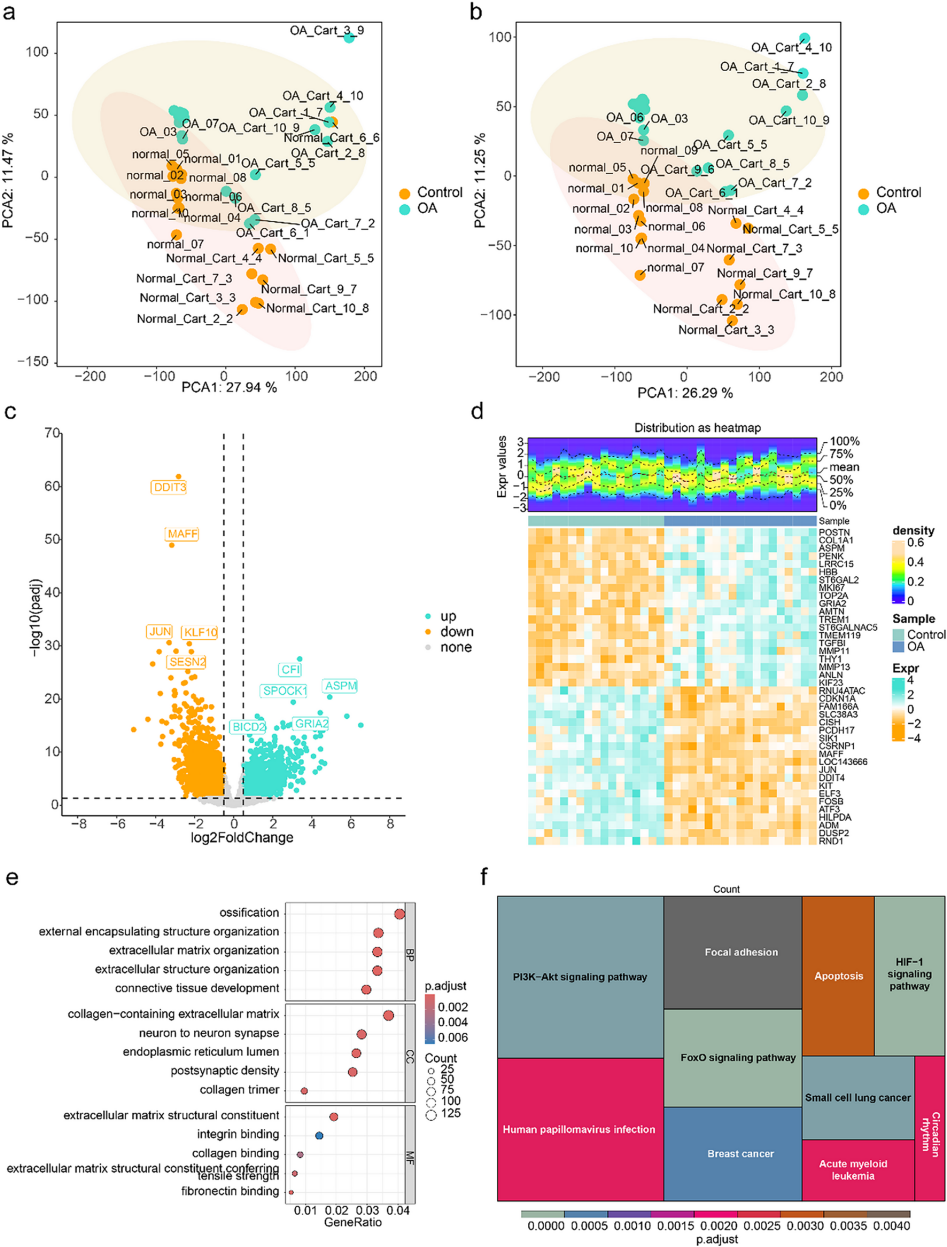
The analysis of different DEGs and multiple signal pathways. (**a-b**) The OA and Control PCA analysis. (**c**) Volcano plot of differential gene analysis. green dots represent upregulated genes, Yellow dots represent downregulated genes, and grey dots represent genes with no significant difference or small fold changes. (**d**) Heatmap of differential gene analysis. The upper half displays a heatmap of expression density, while the lower half shows the expression heatmap. (**e**) The GO differential gene analysis. The horizontal axis represents the enrichment factor, the vertical axis represents the pathway names, the size of the dots indicates the number of enriched genes in the pathway, and the colour represents the range of p-values. (**f**) The KEGG differential gene analysis. The size of the grid represents the number of enriched genes; the color of the grid represents the P-value of the enrichment

The PCA analysis revealed the presence of two outlier samples (OA_Cart_3_9 and normal_Cart_6_6) in the GSE114007 dataset, which were subsequently excluded from further analysis (Fig. 2a-b). A total of 4,178 DEGs were identified between OA and control groups in GSE114007 datasets, containing 2,162 up-regulated genes and 2,016 down-regulated genes (Fig. 2c-d Supplementary Table S2). Furthermore, these DEGs were enriched in extracellular structure organization, neuron to neuron synapse, postsynaptic density, etc. in GO terms (Fig. 2e Supplementary Table S3), and FoxO signaling pathway, apoptosis, HIF-1 signaling pathway, PI3K/Akt signaling pathway, etc. in KEGG pathways (Fig. 2f). Furthermore, enrichment analyses were respectively conducted on the upregulated and downregulated differentially expressed genes (DEGs). The results showed that both the upregulated and downregulated DEGs were enriched in the PI3K/Akt signaling pathway. This finding suggested that in the future, we could explore whether branched-chain amino acids (BCAAs) have an impact on osteoarthritis (OA) through the PI3K/Akt signaling pathway. (Supplementary Table S4)

### SLC3A2 and SLC7A5 were identified as biomarkers

**Fig. 3:**
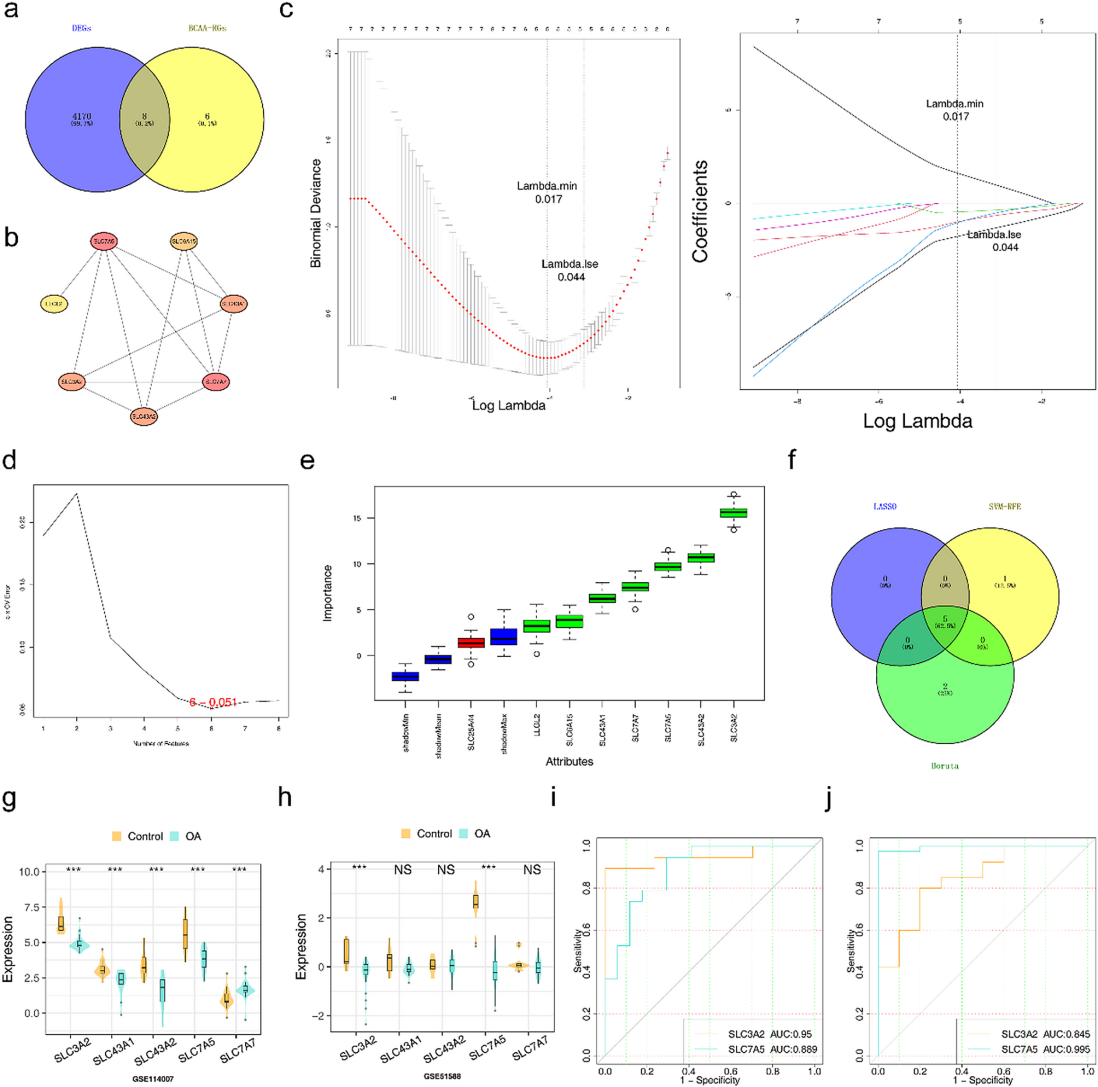
Identification of differential genes. (**a**) Venn diagram of DEGs and BCAA-RGS genes. (**b**) PPI network. (**c-e**) The LASSO regression, SVM-RFE algorithm, and Boruta algorithm coefficient penalty plot. (**f**) Venn diagram of LASSO, SVM-RFE, and Boruta algorithms. (**g-h**) Violin plots visualized the correlation the OA group and control group in datasets. (**i-j**) The ROC curves for risk. ** *P* < 0.01; *** *P* < 0.001

Eight candidate genes were acquired by intersecting 4,178 DEGs and 14 BCAA-RGs (Fig. 3a). PPI results revealed that SLC7A5 interacted with LLGL2, SLC3A2, SLC43A2, SLC7A7 and SLC43A1 (Fig. 3b). In the LASSO regression analysis, the Lambda.min threshold was selected as 0.017. The constructed model had the smallest residual sum of squares, and the corresponding gene and its coefficient were found in the right figure. Finally, 5 characteristic genes were obtained through the LASSO regression analysis. In the SVM-RFE analysis, the results showed that when the number of genes kept increasing, the error rate at the optimal point was 0.051, corresponding to 6 characteristic genes. Through the analysis of the Boruta algorithm, all the feature sets correlated with the dependent variable were screened out, and finally 7 characteristic genes were obtained.(Fig. 3c-e). After taking the intersection of the feature genes obtained from the three machine learning algorithms, five candidate biomarkers were obtained, namely SLC3A2, SLC7A5, SLC43A2, SLC43A1, and SLC7A7 (Fig. 3f). The expression of SLC3A2 and SLC7A5 exhibited a consistent trend in the GSE114007 and GSE51588 datasets, and their expression was significantly higher in the OA group compared to the control group (Fig. 3g-h). Meanwhile, the AUC values of SLC3A2 and SLC7A5 were both greater than 0.7 in the GSE114007 and GSE51588 datasets (Fig. 3i-j); therefore, SLC3A2 and SLC7A5 were identified as biomarkers.

### The nomogram exhibited excellent prediction performance for OA

**Fig. 4:**
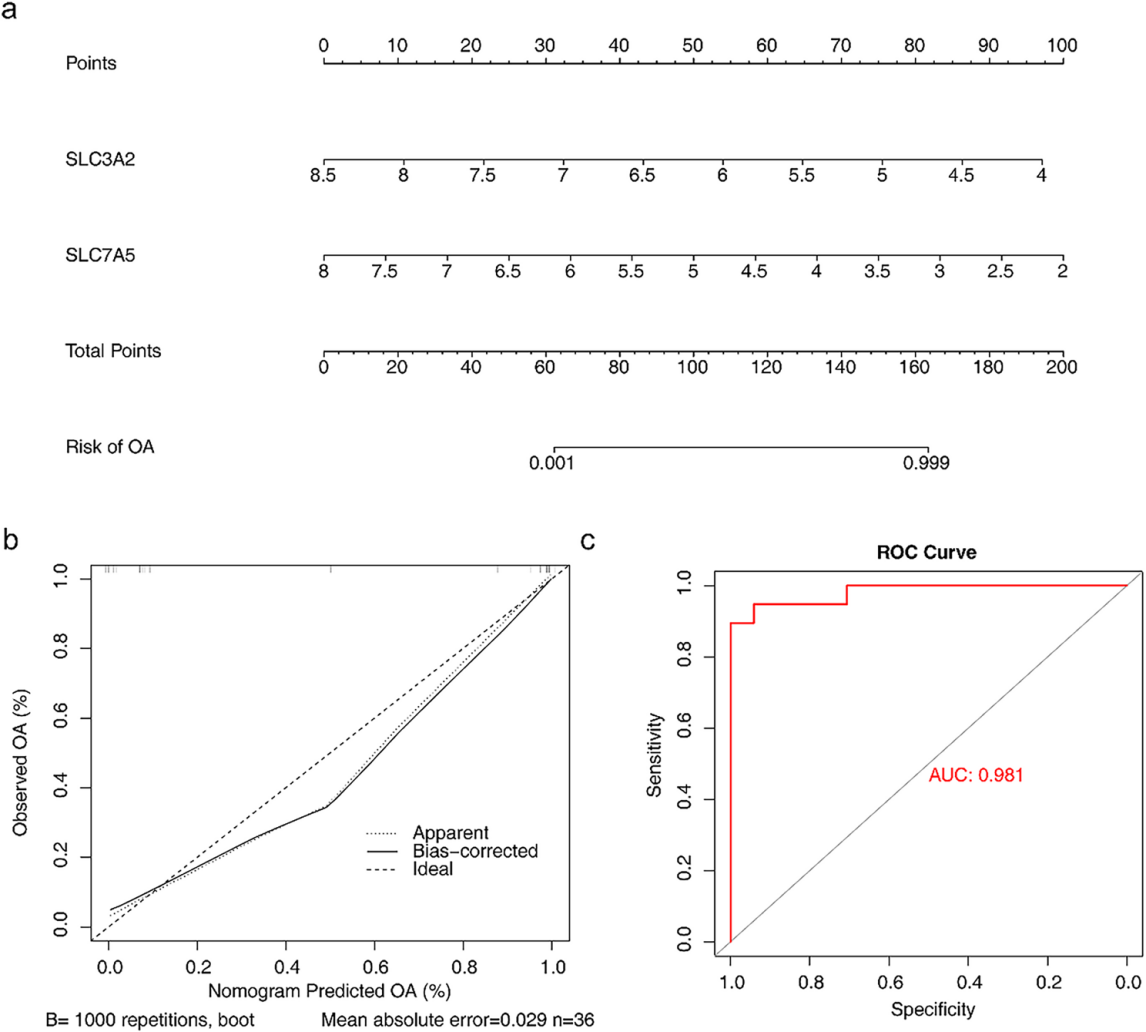
The nomogram exhibited prediction performance for OA. (**a**) Nomogram predicting the incidence. (**b-c**) The calibration curves and ROC adopted to evaluate the accuracy of the predictive power of the nomogram

Based on the biomarkers SLC3A2 and SLC7A5, a nomogram was constructed to assess their predictive ability for OA incidence as a whole (Fig. 4a). Calibration curves and ROC curves were adopted to evaluate the accuracy of the predictive power of the nomogram. Specifically, the slope of the calibration curve tended to be 1 (Fig. 4b), while the AUC value of the nomogram was 0.981 (Fig. 4c). These results suggested that the nomogram exhibited excellent accuracy in predicting the incidence of OA. On this basis, we constructed another nomogram by incorporating clinical characteristics. The results showed that age and gender had certain impacts on the risk of osteoarthritis (OA). Specifically, an age of ≥ 60 years and female (F) gender were likely to correspond to relatively high scores in the assessment of OA risk, suggesting that older age and female gender may be associated factors for OA (Supplementary Fig. S2).

### GSEA enrichment analysis

**Fig. 5:**
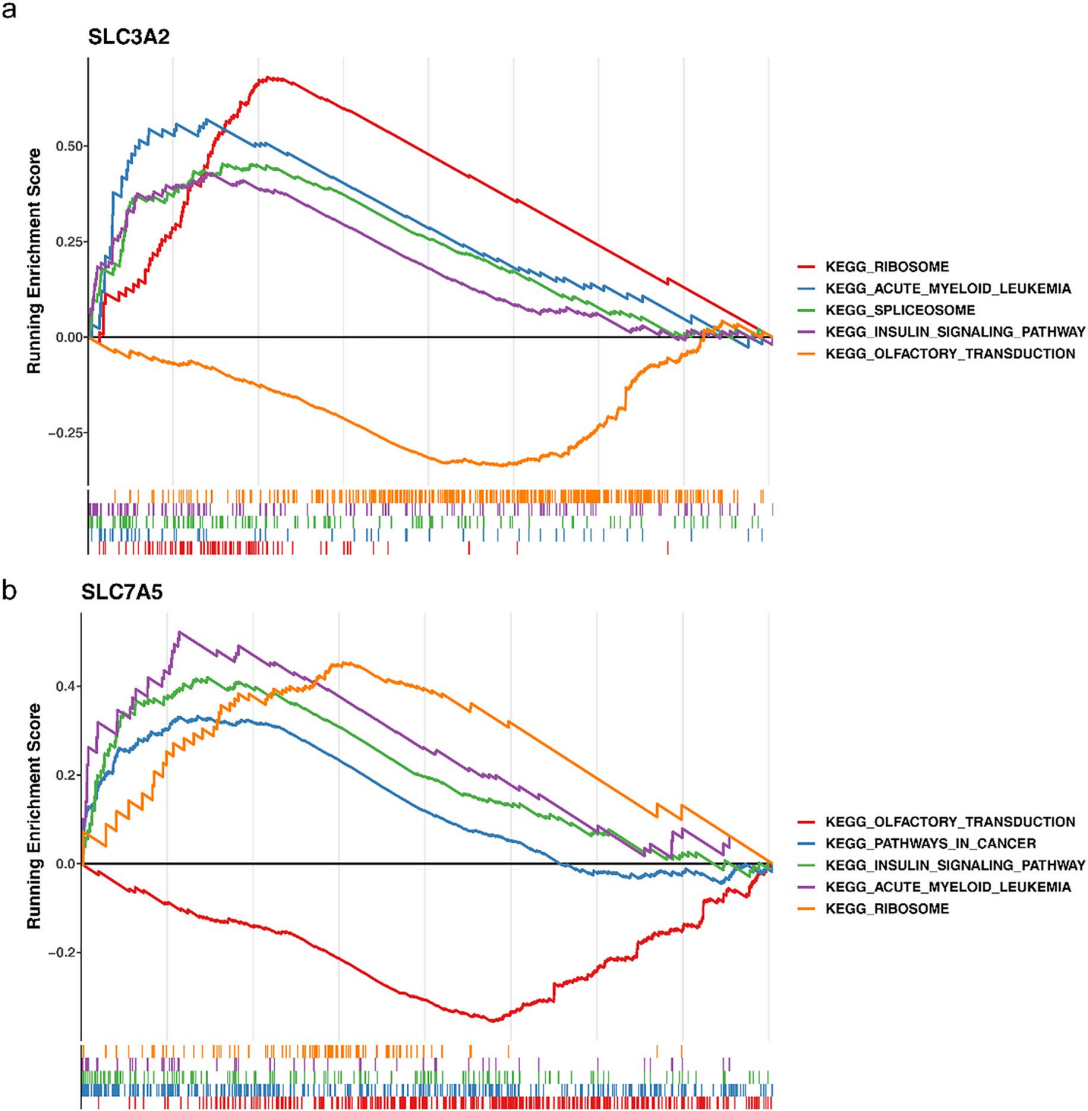
GSEA enrichment analysis. (**a-b**) The SLC3A2 and SLC7A5 KEGG analysis

GSEA was applied to explore some related signaling pathways and potential biological mechanisms of biomarkers. The results demonstrated that SLC3A2 and SLC7A5 were significantly enriched in ribosome, acute myeloid leukemia, insulin signaling pathway, olfactory transduction, etc. (Fig. 5a-b, Supplementary Tables S2–S3).

**SLC3A2 and SLC7A5 were simultaneously associated with multiple regulatory factors and drugs as well as diseases**.

**Fig. 6:**
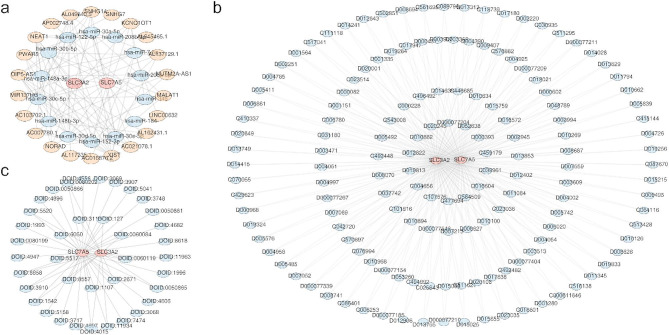
SLC3A2 and SLC7A5 were simultaneously associated with multiple regulatory factors and drugs as well as diseases. (**a**) The lncRNA-miRNA-mRNA network constructed. (**b**) The Acetaminophen and Acrylamide gene-drug regulatory network. (**c**) The Gene-disease association analysis

A total of 14 key miRNAs were predicted based on the analysis of SLC3A2 and SLC7A5. Subsequently, we identified 22 lncRNAs with clipExpNum ≥ 20. Next, a lncRNA-miRNA-mRNA network was constructed, which included 38 nodes and 102 edges. Complex regulatory relationships existed within this network, exemplified by XIST mediated regulation of SLC7A5 via hsa-miR-30e-5p and its regulation of SLC3A2 through hsa-miR-7-5p (Fig. 6a). Furthermore, 150 drugs were identified, including Acetaminophen and Acrylamide, which exhibited simultaneous targeting of these two biomarkers. Consequently, a comprehensive gene-drug regulatory network comprising 152 nodes and 300 edges was constructed (Fig. 6b, Supplementary Table S4). Additionally, the Gene-disease association analysis revealed that these two biomarkers were associated with 38 diseases, such as sarcomatoid carcinoma, colorectal carcinoma, tongue squamous cell carcinoma and so on (Fig. 6c, Supplementary Table S5).

### Expression verification of biomarkers

**Fig. 7:**
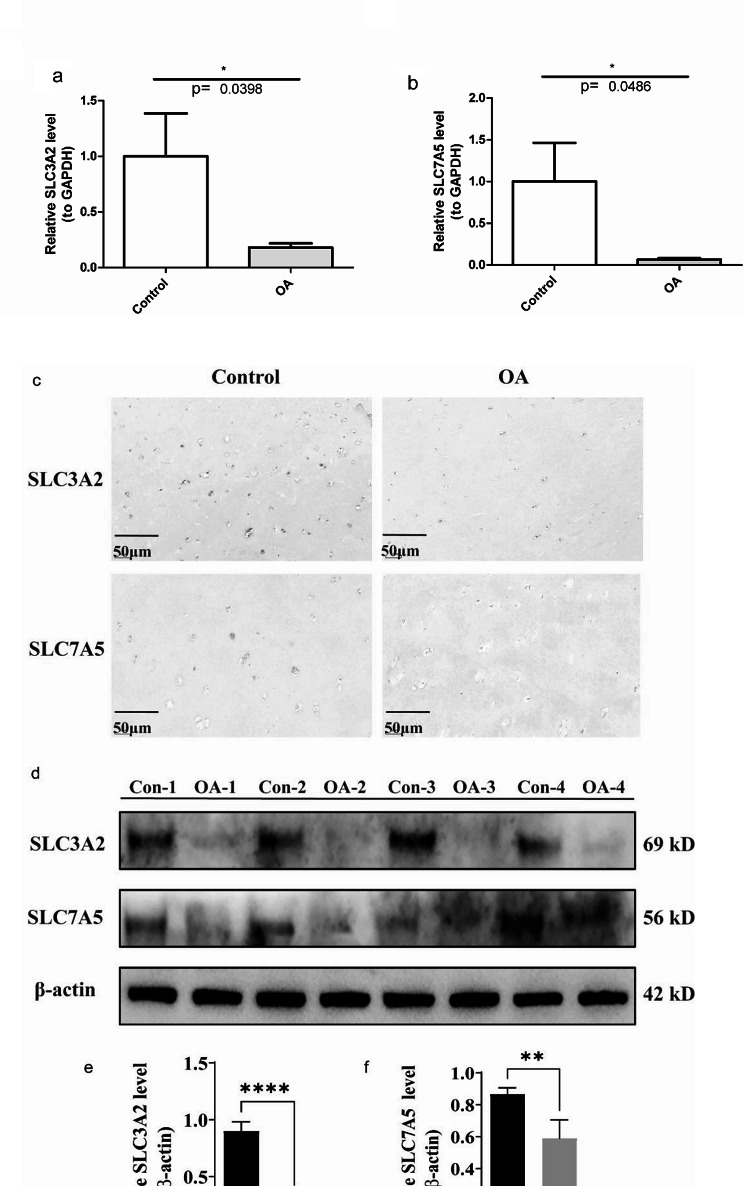
(**a-b**) RT-qPCR analysis of mRNA expression for SLC3A2 and SLC7A5. (**c**) IHC staining of protein levels of SLC3A2 and SLC7A5. (**d**) Western blot analysis of protein levels of SLC3A2 and SLC7A5, full-length gels are presented in supplementary Fig. S1. (**e**) Relative expression level of SLC3A2. (**d**) Relative expression level of SLC7A5. ** *P* < 0.01; *** *P* < 0.001

To determine the expression of biomarkers in osteoarthritis tissues, We detected the expression of SLC3A2 and SLC7A5 in the control group and the OA group by qRT-PCR (Fig. 7a-b). The results showed that the mRNA expressions of SLC3A2 and SLC7A5 in the OA group were significantly lower than those in the control group. Supplementary Fig. S3 showed the gel electrophoresis images of the PCR products for each gene.,. IHC (Fig. 7c) and WB (Fig. 7d-f) results also showed that the protein expression of SLC3A2 and SLC7A5 were significantly decreased in the OA group. These results indicate that the expression levels of SLC3A2 and SLC7A5 are consistent with bioinformatics results.

## Discussion

Osteoarthritis is a chronic joint disease characterized clinically by joint pain, stiffness, and functional impairment. Currently, the main treatments for osteoarthritis include physical therapy, medication, and surgical intervention, but there are problems such as late diagnosis, inconsistent treatment outcomes, and side effects [33, 34]. BCAAs play an important role in the development of osteoarthritis [15, 35]. This study successfully identified two biomarkers associated with BCAAs, namely SLC3A2 and SLC7A5. These two genes are enriched in pathways such as ribosome, acute myeloid leukemia, insulin signaling pathway, and olfactory transduction, suggesting that they may have important roles in the occurrence of OA. By constructing nomogram, we evaluated the combined ability of these two biomarkers for predicting the occurrence of OA. Additionally, we conducted further analyses such as molecular regulatory network and drug prediction to explore the mechanisms of these biomarkers in osteoarthritis. This study primarily relies on the training dataset (GSE114007) for bioinformatics research. The selection of this dataset is based on two aspects: First, the sample types included in this dataset al.ign with our research theme; Second, this dataset has been extensively analyzed in multiple previous osteoarthritis (OA) studies, demonstrating its widespread recognition [36–38]. However, compared to previous studies, our research has some differences. This study combines multiple machine learning algorithms to screen biomarkers, which not only improves the accuracy and reliability of screening but also ensures the robustness of results through external validation and the combination of multiple algorithms.

In this study, we employed LASSO regression, SVM-RFE, and Boruta algorithms to identify key OA biomarkers. LASSO regression effectively reduced feature redundancy but may have missed complex non-linear relationships [39]. Boruta, based on a random forest approach, ensured no relevant features were overlooked, though it risked overfitting due to retaining many features [40]. SVM-RFE accounted for non-linearities but required significant computational resources [41]. The integration of these three machine learning methods allowed us to identify SLC3A2 and SLC7A5 as key biomarkers associated with BCAA metabolism in OA. On this basis, we not only identified key genes related to BCAA metabolism, but also further validated their roles in OA through nomogram and GSEA analysis. The successfully constructed nomogram can be used to predict the occurrence of OA, providing a new tool for clinical diagnosis.

**Fig. 8:**
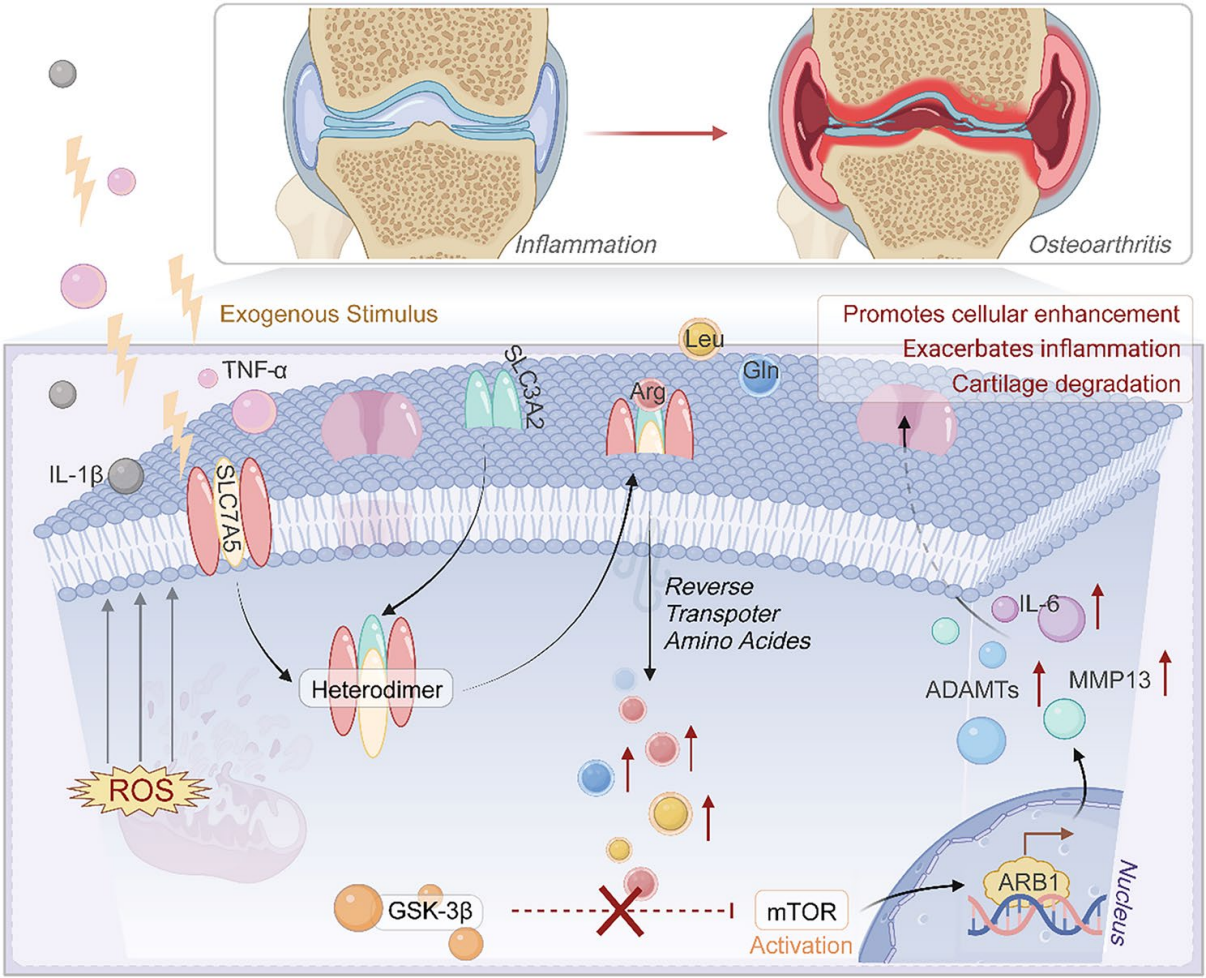
Mechanism of SLC3A2 and SLC7A5 in the pathogenesis of OA. SLC3A2 and SLC7A5 form an amino acid transport heterodimer that facilitates the reverse transport of BCAAs into cytoplasm under the stimulation of extracellular inflammatory factors. The intracellular BCAAs activate the mTOR signaling pathway, which is otherwise suppressed by GSK3β. Activation of mTOR enhances the transcription of ARRB1, leading to the upregulation of IL-6, MMP-9, and ADAMTS expression. This process contributes to increased oxidative stress (ROS). Dashed lines indicate inhibition of enzymatic activity, and the “×” symbol represents pathway blockage

SLC3A2 (solute carrier family 3 member 2) and SLC7A5 (solute carrier family 7 member 5) are identified as biomarkers associated with BCAAs in this study. SLC7A5 and SLC3A2 are both transmembrane glycoproteins that form a heteromeric amino acid transporter (HAT) complex [42]. This complex is responsible for transportation of multiple essential amino acids, including BCAAs [42]. It plays a critical role in protein synthesis and cellular energy metabolism. The SLC7A5/SLC3A2 complex also transports leucine, which can activate the mammalian target of rapamycin complex 1 (mTORC1). Activation of mTORC1 supports cell growth and proliferation [43, 44]. This process is important in various physiological and pathological events, including immune cell regulation, tumor progression, and blood-brain barrier function [45, 46]. Current studies suggested that SLC3A2 and SLC7A5 are significant in various orthopedic diseases. For example, research has indicated that the pathological process of OA cartilage cells is related to ferroptosis, and SLC3A2 inhibits ferroptosis of cartilage cells and suppresses cartilage degeneration in OA [47]. Karouzakis et al. reported that SLC3A2 involved in the onset and development of rheumatoid arthritis (RA) [48]. Furthermore, according to bioinformatics research by Zhao et al., SLC7A5 can serve as a new biomarker for knee arthritis [49]. Based on these findings, we believe that SLC3A2 and SLC7A5 play crucial roles in BCAA metabolism in OA. The underlying mechanism of SLC3A2 and SLC7A5 in the pathogenesis of OA was shown in Fig. 8.

Nomogram is an effective tool for predicting and assessing the probability of clinical events, showing significant advantages over traditional staging systems. It enables individualized prediction and comprehensive multi-factor evaluation, and has been widely applied in clinical practice [50]. Based on biomarkers and related clinical features, this study successfully constructed two nomograms. The nomogram model based on biomarkers demonstrates that SLC3A2 and SLC7A5 have potential diagnostic value in osteoarthritis. Hu et al. identified SLC3A2 as a key regulator of disulfidptosis in OA and developed a predictive, preventive, and personalized medicine (PPPM)-based diagnostic model for OA, in which SLC3A2 showed high accuracy in predicting both early and late stages of the disease [51]. Similarly, Cao et al. highlighted SLC3A2 as hub gene in chondrocyte inflammation and closely related to disulfidptosis through bioinformatics identification [52]. Further experiment study by Jiao et al. showed that Yishen Tongbi decoction, a Chinese traditional herb formula, can suppressed SLC3A2/integrin β3 signaling pathways to inhibit the proliferation and migration of synovial fibroblasts in RA [53]. SLC3A2 also showed potential therapeutic possibility for osteoarthritis, as it inhibited ferroptosis and preventing cartilage degeneration [47]. Our results and previous studied together indicated that SLC3A2 can serve as both therapeutic and diagnostic biomarkers in OA. On the other hand, although SLC7A5 has not been reported as a therapeutic target or predictive biomarker in OA. It had close interaction with SLC3A2, which suggested that it may have similar functional relevance. Considering the established role of SLC3A2 in cartilage protection and metabolic regulation, it is plausible that SLC7A5 also contributes to these processes. In addition, by comparing with existing OA-related nomograms, we found that the nomogram constructed in this study performed better in ROC values compared to existing research [54], indicating that the nomogram constructed in this study has relatively higher predictive value. After incorporating clinical features, we reconstructed the nomogram and found that older age and female gender may be related factors for OA onset. Existing studies have shown that in aging OA joints, senescent cells release senescence-associated mediators, which further damage joint tissues and promote OA development [55]. Epidemiological studies also suggest that the prevalence of osteoarthritis is generally higher in women than in men [56]. Combining these results, our study provides a more accurate basis for early identification of high-risk OA populations, further verifying the key roles of age and gender in OA onset, and offering important references for clinical intervention and prevention strategies.

To further explore the potential biological functions and molecular mechanisms of SLC3A2 and SLC7A5 in OA, GSEA analysis revealed that these genes are enriched in multiple signaling pathways, including the ribosome and insulin signaling pathways. These enrichment results aligned with previous literature on the occurrence and progression of OA. For example, studies have indicated that, in OA, abnormal protein synthesis and metabolic activity may be linked to the dysregulated activation of the ribosome pathway [57, 58]. Additionally, the aberrant activation of the insulin signaling pathway is also associated with the occurrence of osteoarthritis and disturbances in bone metabolism [59, 60]. These findings suggest that the identified biomarkers are involved in these enriched pathways related to OA and may influence disease progression by regulating these pathways.

In addition, we identified several non-coding RNAs associated with osteoarthritis, such as lncRNA OIP5-AS1 and lncRNA XIST. Multiple studies have reported that these non-coding RNAs are associated with the onset and progression of osteoarthritis. For example, OIP5-AS1 has been found to be closely related to inflammatory response, cell apoptosis, and cartilage metabolism. Zhang et al. discovered that OIP5-AS1 inhibited the PI3K/AKT pathway, promoted the survival and proliferation of IL-1β-activated chondrocytes, and prevented apoptosis and extracellular matrix degradation by targeting miR-338-3p [61]. Another study found that OIP5-AS1 was downregulated in human OA tissue and OA animal model, and the downregulation suppressed the proliferation and migration abilities of chondrocyte cells lines through the miR-29b-3p/PGRN axis, thereby promoting chondrocyte apoptosis and inflammation response [62]. Although there was no reports of OIP5-AS1 regulating SLC3A2/SLC7A5 in OA, study have demonstrated that in endometrial carcinoma cells, OIP5-AS1 can upregulate the expression of SLC7A5 by targeting miR-152-3p, thereby promoting cell proliferation [63]. This finding suggested that OIP5-AS1 may potentially regulate the expression of the SLC3A2/SLC7A5 complex, although the specific mechanisms still require further investigation. X-inactive-specific transcript (XIST), a common oncogene in various cancers, was reported to be aberrantly upregulated in OA tissue specimens and generated the pro-inflammatory effect [64], indicating the involvement of XIST in OA pathogenicity. Besides, it has been reported that miR-149 was down regulated in OA chondrocytes and related to inflammation in OA [65]. Another study reported that down-regulated XIST was involved in the injury of chondrocytes during the pathophysiological process of OA, and XIST up-regulation protected chondrocytes from inflammatory injury via regulating miR-653-5p/SIRT1 axis [66].These regulatory network analysis results reveal the important regulatory roles of non-coding RNAs in the pathogenesis of osteoarthritis.

According to drug prediction analysis, we found that Acetaminophen and acrylamide may be closely related to SLC3A2 and SLC7A5. These drugs or substances may have certain relevance to the treatment or pathogenesis of osteoarthritis. For example, Acetaminophen is widely used to alleviate pain in osteoarthritis patients, mainly by inhibiting pain-related pathways [67–69]. Although there is currently a lack of direct evidence proving the association between Acrylamide and osteoarthritis, there is research suggesting that Acetaminophen may increase the risk of osteoporotic fractures in osteoarthritis patients [70, 71]. The potential regulatory mechanisms of these drugs or substances are consistent with the biomarkers and pathways we identified in our study, providing a reference for future clinical treatments.

This study utilized multiple bioinformatics and machine learning approaches, which enhanced the accuracy of identifying BCAA-related genes in OA. The results provided new references for the diagnosis and treatment of OA. Further validation the expression of SLC3A2 and SLC7A5 through qRT-PCR and immunohistochemistry added credibility to our findings. However, this study has several limitations. First, it relies on transcriptome data from the Gene Expression Omnibus (GEO) database, which has limited sample size and lacks validation in independent cohorts. Moreover, our analysis did not deeply explore the potential mechanisms of SLC3A2 and SLC7A5. Another limitation is that the samples used in this study are limited to cartilage tissue. In the construction of the ceRNA network, we were unable to strictly analyze based on the ceRNA hypothesis, which to some extent affected the credibility of the final results. Future studies will use larger and more representative datasets for analysis and validation, and plan to reconstruct the network based on the ceRNA hypothesis. Meanwhile, we will consider using centrality measurement methods to identify core genes in the network, thereby revealing potential key regulatory axes to enhance the biological significance of the network. Based on this, we also plan to conduct in vitro and in vivo functional studies, as well as comparative analysis of multiple tissue types. In addition, preclinical drug testing will be conducted to further understand the roles of these biomarkers and evaluate their therapeutic potential.

## Conclusions

In conclusion, this study, using bioinformatics methods, has discovered biomarkers associated with BCAAs and constructed line graphs, yielding innovative and remarkable results. Although further validation and experimental research are still needed, this study has laid the groundwork for exploring the mechanism of OA and developing personalized treatment approaches. The research provides new directions and strategies for the prevention, early diagnosis, and treatment of OA, aiming to improve the quality of life for patients and alleviate the societal economic burden.

## Electronic supplementary material

Below is the link to the electronic supplementary material.

Supplementary Figure 1: The full-length gels.

Supplementary Figure 2: Construction of a nomogram based on key genes and clinical characteristics and its related validations(a) Nomogram constructed based on key genes and clinical characteristics.(b - c) Calibration curves and receiver - operating characteristic (ROC) curves used to assess the accuracy of the nomogram’s predictive ability.

Supplementary Figure 3: The gel electrophoresis images of the PCR products for each gene.

Supplementary Table 1: The primer sequences for PCR.

Supplementary Table 2: Results of differential gene analysis.

Supplementary Table 3: Differentially expressed genes based on Gene Ontology (GO) enrichment analysis.

Supplementary Table 4: Results of enrichment analysis of up- and down-regulated differentially expressed genes.

Supplementary Table 5-6: GSEA enrichment analysis.

Supplementary Table 7: The 150 drugs were identified.

Supplementary Table 8: The Gene-disease association analysis, two biomarkers were associated with 38 diseases.

## Data Availability

The data analyzed in this research were collected from the Gene Expression Omnibus (GEO) (http://www.ncbi.nlm.nih.gov/geo/) database. further inquiries can be directed to the corresponding author.

## Abbreviations

BCAA: Branched-chain amino acids
OA: Osteoarthritis
BCAA-RGs: BCAA related genes
GEO: Gene Expression Omnibus
GO: Gene Ontology
KEGG: Kyoto Encyclopedia of Genes and Genomes
ROCs: Receiver operating characteristic curves
DEGs: Differentially expressed genes
qRT-PCR: Quantitative real-time polymerase chain reaction
GSEA: Genome Enrichment Analysis
MSigDB: Molecular Signatures Database
PCA: Principal component analysis
PPI: Protein-protein interaction
LASSO: Least absolute shrinkage and selection operator
SVM-RFE: Support vector machine recursive feature elimination
ROC: Receiver operating characteristic
AUC: Area under the curve
GSEA: Gene set enrichment analysis
NES: Normalized enrichment score
lncRNAs: Long noncoding RNAs
CTD: Comparative Toxicogenomics Database

## References

[1] Abramoff B, Caldera FE. Osteoarthritis: pathology, diagnosis, and treatment options. Med Clin North Am. 2020;104(2):293–311. 32035570 10.1016/j.mcna.2019.10.007

[2] Bliddal H. [Definition, pathology and pathogenesis of osteoarthritis]. Ugeskr Laeger, 2020. 182(42). 33046193

[3] Zhu S, et al. Chinese guidelines for the rehabilitation treatment of knee osteoarthritis: an CSPMR evidence-based practice guideline. J Evid Based Med. 2023;16(3):376–93. 37743650 10.1111/jebm.12555

[4] Yao Q, et al. Osteoarthritis: pathogenic signaling pathways and therapeutic targets. Signal Transduct Target Ther. 2023;8(1):56. 36737426 10.1038/s41392-023-01330-w PMC9898571

[5] Motta F, et al. Inflammaging and osteoarthritis. Clin Rev Allergy Immunol. 2023;64(2):222–38. 35716253 10.1007/s12016-022-08941-1

[6] Mehana EE, Khafaga AF, El-Blehi SS. The role of matrix metalloproteinases in osteoarthritis pathogenesis: an updated review. Life Sci. 2019;234:116786. 31445934 10.1016/j.lfs.2019.116786

[7] Litwic A, et al. Epidemiology and burden of osteoarthritis. Br Med Bull. 2013;105:185–99. 23337796 10.1093/bmb/lds038 PMC3690438

[8] Scheuing WJ, et al. The burden of osteoarthritis: is it a rising problem? Best Pract Res Clin Rheumatol. 2023;37(2):101836. 37633827 10.1016/j.berh.2023.101836

[9] Salman LA, et al. Osteoarthritis: a narrative review of molecular approaches to disease management. Arthritis Res Ther. 2023;25(1):27. 36800974 10.1186/s13075-023-03006-w PMC9938549

[10] Emami A, et al. Challenges in osteoarthritis treatment. Tissue Cell. 2023;80:101992. 36462384 10.1016/j.tice.2022.101992

[11] Basavanna S, et al. Screening of Streptococcus pneumoniae ABC transporter mutants demonstrates that LivJHMGF, a branched-chain amino acid ABC transporter, is necessary for disease pathogenesis. Infect Immun. 2009;77(8):3412–23. 19470745 10.1128/IAI.01543-08 PMC2715661

[12] Le Couteur DG, et al. Branched chain amino acids, aging and age-related health. Ageing Res Rev. 2020;64:101198. 33132154 10.1016/j.arr.2020.101198

[13] Neinast M, Murashige D, Arany Z. Branched Chain Amino Acids Annu Rev Physiol. 2019;81:139–64. 30485760 10.1146/annurev-physiol-020518-114455 PMC6536377

[14] Nie C et al. Branched chain amino acids: beyond nutrition metabolism. Int J Mol Sci, 2018. 19(4). 10.3390/ijms19040954 PMC5979320 29570613

[15] Zhai G, et al. Serum branched-chain amino acid to histidine ratio: a novel metabolomic biomarker of knee osteoarthritis. Ann Rheum Dis. 2010;69(6):1227–31. 20388742 10.1136/ard.2009.120857

[16] Liu M et al. Restricting Branched-Chain amino acids within a High-Fat diet prevents obesity. Metabolites, 2022. 12(4). 10.3390/metabo12040334 PMC9030079 35448521

[17] White PJ, et al. Insulin action, type 2 diabetes, and branched-chain amino acids: A two-way street. Mol Metab. 2021;52:101261. 34044180 10.1016/j.molmet.2021.101261 PMC8513145

[18] Kapoor M, et al. Role of Proinflammatory cytokines in the pathophysiology of osteoarthritis. Nat Rev Rheumatol. 2011;7(1):33–42. 21119608 10.1038/nrrheum.2010.196

[19] Jamshidi A, Pelletier JP, Martel-Pelletier J. Machine-learning-based patient-specific prediction models for knee osteoarthritis. Nat Rev Rheumatol. 2019;15(1):49–60. 30523334 10.1038/s41584-018-0130-5

[20] Arbeeva L, et al. Machine learning approaches to the prediction of osteoarthritis phenotypes and outcomes. Curr Rheumatol Rep. 2023;25(11):213–25. 37561315 10.1007/s11926-023-01114-9 PMC10592147

[21] Love MI, Huber W, Anders S. Moderated Estimation of fold change and dispersion for RNA-seq data with DESeq2. Genome Biol. 2014;15(12):550. 25516281 10.1186/s13059-014-0550-8 PMC4302049

[22] Gustavsson EK, et al. Ggtranscript: an R package for the visualization and interpretation of transcript isoforms using ggplot2. Bioinformatics. 2022;38(15):3844–6. 35751589 10.1093/bioinformatics/btac409 PMC9344834

[23] Gu Z, Eils R, Schlesner M. Complex heatmaps reveal patterns and correlations in multidimensional genomic data. Bioinformatics. 2016;32(18):2847–9. 27207943 10.1093/bioinformatics/btw313

[24] Yu G, et al. ClusterProfiler: an R package for comparing biological themes among gene clusters. OMICS. 2012;16(5):284–7. 22455463 10.1089/omi.2011.0118 PMC3339379

[25] Li Y, Lu F, Yin Y. Applying logistic LASSO regression for the diagnosis of atypical Crohn’s disease. Sci Rep. 2022;12(1):11340. 35790774 10.1038/s41598-022-15609-5 PMC9256608

[26] Hu X, et al. Combining metabolome and clinical indicators with machine learning provides some promising diagnostic markers to precisely detect smear-positive/negative pulmonary tuberculosis. BMC Infect Dis. 2022;22(1):707. 36008772 10.1186/s12879-022-07694-8 PMC9403968

[27] Twait EL, et al. Dementia prediction in the general population using clinically accessible variables: a proof-of-concept study using machine learning. The AGES-Reykjavik study. BMC Med Inf Decis Mak. 2023;23(1):168. 10.1186/s12911-023-02244-x PMC10463542 37641038

[28] Robin X, et al. pROC: an open-source package for R and S + to analyze and compare ROC curves. BMC Bioinformatics. 2011;12:77. 21414208 10.1186/1471-2105-12-77 PMC3068975

[29] Xu J, et al. A nomogram for predicting prognosis of patients with cervical cerclage. Heliyon. 2023;9(11):e21147. 37885715 10.1016/j.heliyon.2023.e21147 PMC10598483

[30] Wang L, et al. Cuproptosis related genes associated with Jab1 shapes tumor microenvironment and Pharmacological profile in nasopharyngeal carcinoma. Front Immunol. 2022;13:989286. 36618352 10.3389/fimmu.2022.989286 PMC9816571

[31] Shannon P, et al. Cytoscape: a software environment for integrated models of biomolecular interaction networks. Genome Res. 2003;13(11):2498–504. 14597658 10.1101/gr.1239303 PMC403769

[32] Yu G, et al. DOSE: an R/Bioconductor package for disease ontology semantic and enrichment analysis. Bioinformatics. 2015;31(4):608–9. 25677125 10.1093/bioinformatics/btu684

[33] Tuncay Duruoz M, et al. Clinical aspects and outcomes in osteoarthritis. Best Pract Res Clin Rheumatol. 2023;37(2):101855. 37524622 10.1016/j.berh.2023.101855

[34] Rossbach P. [Osteoarthritis - Therapy and management]. Ther Umsch. 2023;80(1):11–5. 36659844 10.1024/0040-5930/a001401

[35] Zhang W, et al. Metabolomic analysis of human plasma reveals that arginine is depleted in knee osteoarthritis patients. Osteoarthritis Cartilage. 2016;24(5):827–34. 26708258 10.1016/j.joca.2015.12.004

[36] He M, et al. Screening and validation of key genes associated with osteoarthritis. BMC Musculoskelet Disord. 2024;25(1):954. 39587568 10.1186/s12891-024-08015-7 PMC11587628

[37] Song C, et al. Identification of ferroptosis-related genes and potential drugs in osteoarthritis. Inflamm Res. 2025;74(1):70. 40299032 10.1007/s00011-025-02040-5

[38] Yuan WH, et al. Screening of osteoarthritis diagnostic markers based on immune-related genes and immune infiltration. Sci Rep. 2021;11(1):7032. 33782454 10.1038/s41598-021-86319-7 PMC8007625

[39] Zhang L et al. Lasso regression: from explanation to prediction. Adv Psychol Sci, 2020. 28.

[40] Syed Mustapha SMFD. Predictive analysis of students’ learning performance using data mining techniques: A comparative study of feature selection methods. 2023. 6(5): p. 86.

[41] Lin X et al. Selecting feature subsets based on SVM-RFE and the overlapping ratio with applications in bioinformatics. 2018. 23(1): p. 52. 10.3390/molecules23010052 PMC5943966 29278382

[42] Kahlhofer J, Teis D. The human LAT1-4F2hc (SLC7A5-SLC3A2) transporter complex: physiological and pathophysiological implications. Basic Clin Pharmacol Toxicol. 2023;133(5):459–72. 36460306 10.1111/bcpt.13821 PMC11497297

[43] Sokolov AM, Feliciano DM. Slc7a5 regulation of neural development. Neural Regen Res. 2021;16(10):1994–5. 33642374 10.4103/1673-5374.308086 PMC8343322

[44] Sokolov AM, Holmberg JC, Feliciano DM. The amino acid transporter Slc7a5 regulates the mTOR pathway and is required for granule cell development. Hum Mol Genet. 2020;29(18):3003–13. 32821949 10.1093/hmg/ddaa186 PMC7645712

[45] Nachef M, et al. Targeting SLC1A5 and SLC3A2/SLC7A5 as a potential strategy to strengthen Anti-Tumor immunity in the tumor microenvironment. Front Immunol. 2021;12:624324. 33953707 10.3389/fimmu.2021.624324 PMC8089370

[46] Song W, et al. Solute carrier transporters: the metabolic gatekeepers of immune cells. Acta Pharm Sin B. 2020;10(1):61–78. 31993307 10.1016/j.apsb.2019.12.006 PMC6977534

[47] Liu H et al. Identification of SLC3A2 as a potential therapeutic target of osteoarthritis involved in ferroptosis by integrating bioinformatics, clinical factors and experiments. Cells, 2022. 11(21). 10.3390/cells11213430 PMC9657506 36359826

[48] Karouzakis E, et al. Increased recycling of polyamines is associated with global DNA hypomethylation in rheumatoid arthritis synovial fibroblasts. Arthritis Rheum. 2012;64(6):1809–17. 22170508 10.1002/art.34340

[49] Zhao Y et al. Cross-Tissue Analysis Using Machine Learning to Identify Novel Biomarkers for Knee Osteoarthritis. Comput Math Methods Med, 2022. 2022: p. 9043300. 10.1155/2022/9043300 PMC9246600 35785145

[50] Akechi T, et al. Clinical practice guidelines for the care of psychologically distressed bereaved families who have lost members to physical illness including cancer. Jpn J Clin Oncol. 2022;52(6):650–3. 35253040 10.1093/jjco/hyac025 PMC9157299

[51] Hu K, et al. Identification and construction of a Disulfidptosis-Mediated diagnostic model and associated immune microenvironment of osteoarthritis from the perspective of PPPM. J Inflamm Res. 2024;17:3753–70. 38882183 10.2147/JIR.S462179 PMC11179642

[52] Cao S et al. Bioinformatics identification and experimental verification of Disulfidptosis-Related genes in the progression of osteoarthritis. Biomedicines, 2024. 12(8). 10.3390/biomedicines12081840 PMC11351109 39200304

[53] Jiao W, et al. Anti-proliferation and anti-migration effects of Yishen Tongbi Decoction in experimental rheumatoid arthritis by suppressing SLC3A2/integrin Β3 signaling pathways. Phytomedicine. 2023;114:154741. 36990010 10.1016/j.phymed.2023.154741

[54] Chen Q, et al. Integrative bioinformatics analysis reveals novel insights into osteoarthritis pathogenesis and diagnostic biomarkers. BMC Musculoskelet Disord. 2024;25(1):999. 39639239 10.1186/s12891-024-08124-3 PMC11619307

[55] O’Brien MS, McDougall JJ. Age and frailty as risk factors for the development of osteoarthritis. Mech Ageing Dev. 2019;180:21–8. 30898635 10.1016/j.mad.2019.03.003

[56] Pan Q, et al. Characterization of Osteoarthritic human knees indicates potential sex differences. Biol Sex Differ. 2016;7:27. 27257472 10.1186/s13293-016-0080-z PMC4890516

[57] van den Akker GGH, et al. Ribosome dysfunction in osteoarthritis. Curr Opin Rheumatol. 2022;34(1):61–7. 34750309 10.1097/BOR.0000000000000858 PMC8638817

[58] Ripmeester EGJ, et al. Impaired chondrocyte U3 snorna expression in osteoarthritis impacts the chondrocyte protein translation apparatus. Sci Rep. 2020;10(1):13426. 32778764 10.1038/s41598-020-70453-9 PMC7417995

[59] Xu B, et al. Roles of MicroRNA and signaling pathway in osteoarthritis pathogenesis. J Zhejiang Univ Sci B. 2016;17(3):200–8. 26984840 10.1631/jzus.B1500267 PMC4794511

[60] Xue F, et al. Analysis of critical molecules and signaling pathways in osteoarthritis and rheumatoid arthritis. Mol Med Rep. 2013;7(2):603–7. 23232804 10.3892/mmr.2012.1224

[61] Zhang X, et al. LncRNA OIP5-AS1 attenuates the osteoarthritis progression in IL-1beta-stimulated chondrocytes. Open Med (Wars). 2023;18(1):20230721. 37333451 10.1515/med-2023-0721 PMC10276615

[62] Zhi L, et al. Downregulation of LncRNA OIP5-AS1 induced by IL-1beta aggravates osteoarthritis via regulating miR-29b-3p/PGRN. Cartilage. 2021;13(2suppl):S1345–55. 10.1177/1947603519900801 PMC8804817 32037864

[63] Liang M, et al. OIP5-AS1 contributes to the development in endometrial carcinoma cells by targeting miR-152-3p to up-regulate SLC7A5. Cancer Cell Int. 2021;21(1):440. 34419049 10.1186/s12935-021-02061-0 PMC8379738

[64] Li L, et al. XIST/miR-376c-5p/OPN axis modulates the influence of Proinflammatory M1 macrophages on osteoarthritis chondrocyte apoptosis. J Cell Physiol. 2020;235(1):281–93. 31215024 10.1002/jcp.28968

[65] Santini P, et al. The inflammatory circuitry of miR-149 as a pathological mechanism in osteoarthritis. Rheumatol Int. 2014;34(5):711–6. 23595570 10.1007/s00296-013-2754-8

[66] Lian LP, Xi XY. Long non-coding RNA XIST protects chondrocytes ATDC5 and CHON-001 from IL-1beta-induced injury via regulating miR-653-5p/SIRT1 axis. J Biol Regul Homeost Agents. 2020;34(2):379–91. 32517436 10.23812/19-549-A-65

[67] Towheed TE, et al. Acetaminophen for osteoarthritis. Cochrane Database Syst Rev. 2006;2006(1):CD004257. 16437479 10.1002/14651858.CD004257.pub2 PMC8275921

[68] Towheed TE et al. Acetaminophen for osteoarthritis. Cochrane Database Syst Rev, 2003(2): p. CD004257. 10.1002/14651858.CD004257 12804508

[69] D’Arcy Y, et al. Treating osteoarthritis pain: mechanisms of action of acetaminophen, nonsteroidal anti-inflammatory drugs, opioids, and nerve growth factor antibodies. Postgrad Med. 2021;133(8):879–94. 34252357 10.1080/00325481.2021.1949199

[70] Zhao FC, et al. Relationship between acrylamide and glycidamide hemoglobin adduct levels and osteoarthritis: a NHANES analysis. Environ Sci Pollut Res Int. 2023;30(30):75262–72. 37213021 10.1007/s11356-023-27515-y

[71] Veronese N, et al. Dietary acrylamide and incident osteoporotic fractures: an 8-year prospective cohort study. Aging Clin Exp Res. 2022;34(10):2441–8. 35962898 10.1007/s40520-022-02214-9 PMC9637630

