# Supplementary figures and images for: Integrating bioinformatics and machine learning to identify biomarkers of branched chain amino acid related genes in osteoarthritis
Source: BMC Musculoskelet Disord. 2025 May 26;26:517. doi: 10.1186/s12891-025-08779-6 (PMC12105201; doi:10.1186/s12891-025-08779-6)

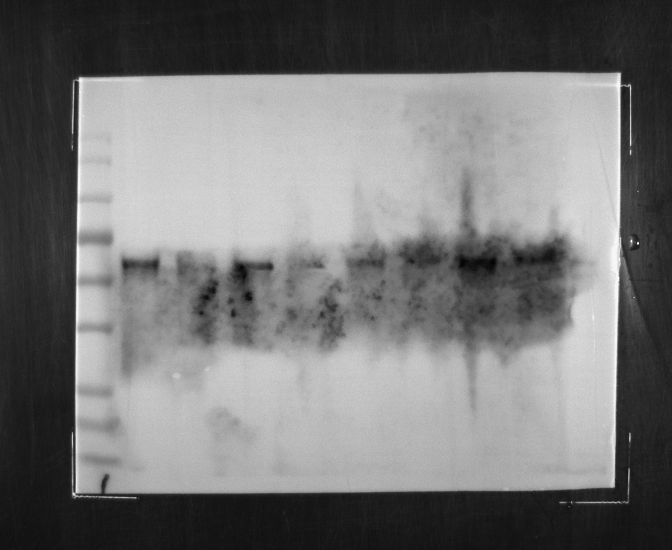

Supplement: Supplementary file 1 — Supplementary Figure 1: The full-length gels. [file 12891_2025_8779_MOESM1_ESM.tif]

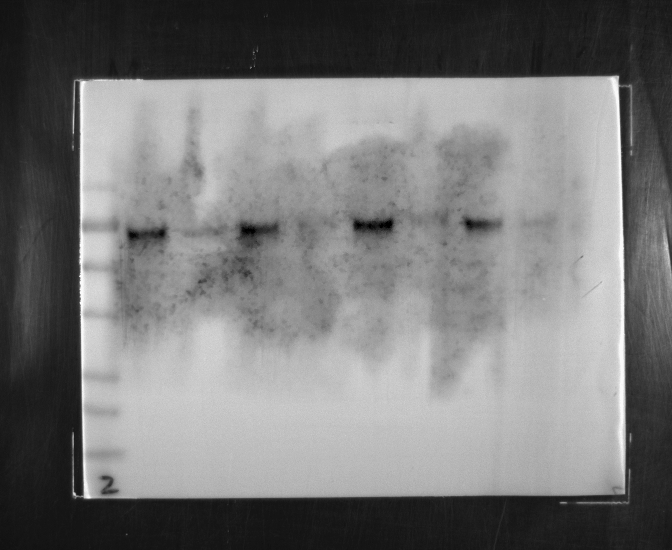

Supplement: Supplementary file 2 — Supplementary Figure 2: Construction of a nomogram based on key genes and clinical characteristics and its related validations(a) Nomogram constructed based on key genes and clinical characteristics.(b - c) Calibration curves and receiver - operating characteristic (ROC) curves used to assess the accuracy of the nomogram’s predictive ability. [file 12891_2025_8779_MOESM2_ESM.tif]

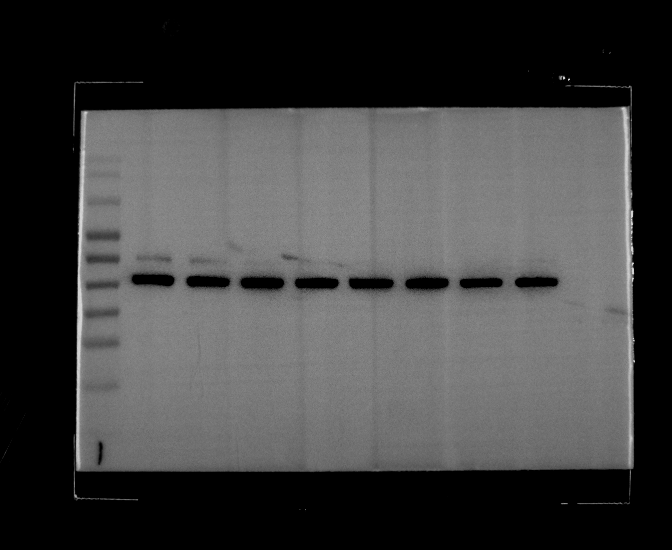

Supplement: Supplementary file 3 — Supplementary Figure 3: The gel electrophoresis images of the PCR products for each gene. [file 12891_2025_8779_MOESM3_ESM.tif]

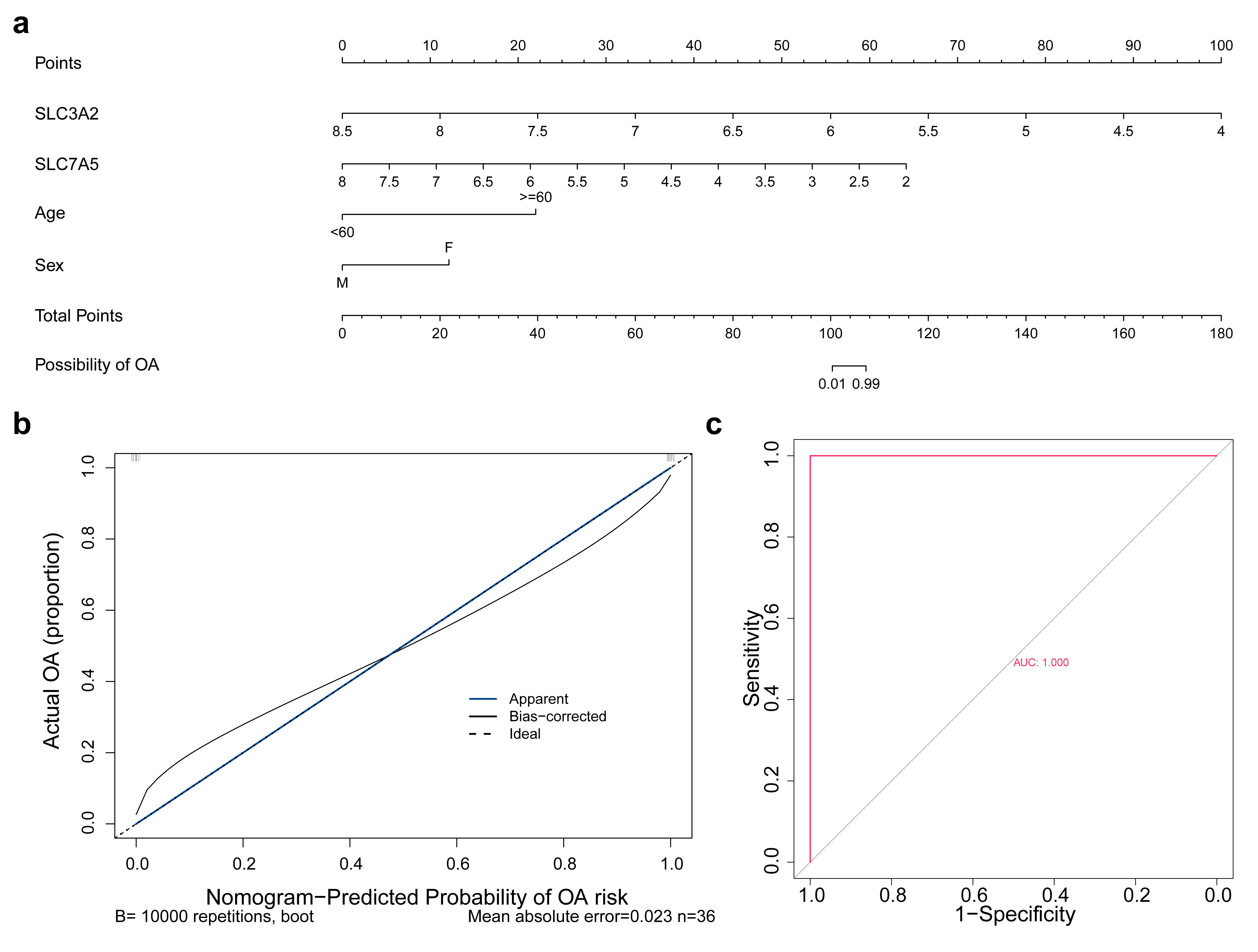

Supplement: Supplementary file 4 — Supplementary Table 1: The primer sequences for PCR. [file 12891_2025_8779_MOESM4_ESM.tif]

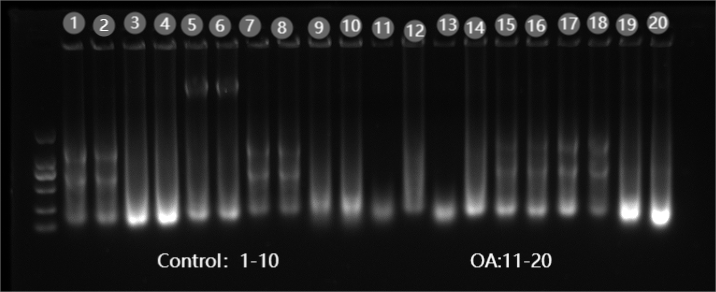

Supplement: Supplementary file 5 — Supplementary Table 2: Results of differential gene analysis. [file 12891_2025_8779_MOESM5_ESM.tif]
