# Supplementary material for: Integrating bioinformatics and machine learning to identify biomarkers of branched chain amino acid related genes in osteoarthritis
Source: BMC Musculoskelet Disord. 2025 May 26;26:517. doi: 10.1186/s12891-025-08779-6 (PMC12105201; doi:10.1186/s12891-025-08779-6)
Supplement: Supplementary file 6 — Supplementary Table 3: Differentially expressed genes based on Gene Ontology (GO) enrichment analysis. [file 12891_2025_8779_MOESM6_ESM.docx]

| **Primer** | **Sequences** | |
| --- | --- | --- |
| SLC3A2 F | AGCTGGAGTTTGTCTCAGGC | |
| SLC3A2 R | AGAAGGGTGTGCTGTTTGGAG |  |
| SLC7A5 F | CCGTGAACTGCTACAGCGTG |  |
| SLC7A5 R | AGGCCGCTGTATAATGCCAG |  |
| 内参-GAPDH F | CGAAGGTGGAGTCAACGGATTT |  |
| 内参-GAPDH R | ATGGGTGGAATCATATTGGAAC |  |
